# A review of patient questions from physicist—patient consults

**DOI:** 10.1002/acm2.12942

**Published:** 2020-06-09

**Authors:** Todd F. Atwood, Derek W. Brown, Titania Juang, Kevin L. Moore, Kristen A. McConnell, Jennifer M. Steers, James D. Murphy, Arno J. Mundt, Todd Pawlicki

**Affiliations:** ^1^ Department of Radiation Medicine & Applied Sciences UC San Diego La Jolla CA USA

**Keywords:** patient communication, patient education, patient questions, physicist–patient consults

## Abstract

**Purpose:**

To provide insight into the types of questions asked to medical physicists by patients during one‐on‐one physicist–patient consults at one institution.

**Materials and Methods:**

Medical physicists trained in patient communication techniques met with patients to provide an overview of the treatment planning and delivery processes, discuss the patient's treatment plan, and answer any technical questions. From August 2016 to December 2019, 152 physicist–patient consults were conducted. In the initial months of the study (August 2016—December 2017), following each physicist–patient consult, all patient questions were documented by the physicists. For the remaining time period (January 2018—December 2019), any newly encountered questions were periodically added to the list. The questions were compiled into a comprehensive list and organized into categories.

**Results:**

There were a total of 88 unique patient questions. These questions fit into four topical categories. Fifty‐four questions (61.4%) were in the “Treatment Planning and Delivery Questions” category, 15 questions (17.1%) were in the “General Radiation Questions or Concerns” category, 13 questions (14.8%) were in the “Safety and Quality Assurance Questions” category, and 6 questions (6.8%) were in the “Medical Questions” category. Overall, patients were primarily concerned about how radiation works, the treatment planning and delivery processes, and what is being done to keep them safe throughout their treatment.

**Conclusion:**

Physicist–patient consults provided an opportunity to address the technical aspects of radiation therapy with patients in greater detail. The fact that patient questions could be conveniently grouped into only four topical categories indicates that it may be straightforward for other medical physicists to prepare for effectively addressing technical questions during physicist–patient consults.

## 1 INTRODUCTION

Patients often view radiation therapy as a technically complex and confusing medical specialty. Common misconceptions and preconceived notions about radiation and radiation therapy frequently result in patients having multiple questions about the medical and technical aspects of their treatment.^1^ This will likely continue as radiation oncology patients become more involved in their care or look for answers online.^2^, ^3^

Studies have shown that information available online is often too complex or convoluted for the general public.^4^, ^5^, ^6^ Regardless of this evidence, a recent study concluded that the readability levels of newly created patient education materials for radiation oncology are not improving on a consistent basis.^7^ Therefore, it is likely that patients will continue to have unresolved questions and concerns about their treatment. At the same time, confusion or frustration around unanswered questions can contribute to patient‐related distress, which may negatively impact outcomes following radiation therapy.^8^

There have been efforts to help address some of these issues by bringing physicists into the patient consultation process.^9^, ^10^ Physicist–patient consults, where medical physicists establish an independent professional relationship with patients to discuss the technical aspects of their radiation therapy, have been shown to be effective in providing accurate information to patients, reducing their anxiety, and increasing their satisfaction.^11^ Offering patients consultations with physicists may increase their level of understanding and therefore their autonomy.^12^

The purpose of this work is to provide insight into the types of questions asked to medical physicists by patients during one‐on‐one physicist–patient consults at one institution.

## 2 MATERIALS AND METHODS

Four medical physicists completed a comprehensive training program to participate in physicist–patient consults.^13^ This training included a physician‐led one day clinician–patient communication workshop run by the medical school, physicist‐to‐physicist practice sessions, simulated patient interactions with trained actors, and faculty‐observed patient consults. Nine patient communication competencies were assessed for all participating medical physicists, each scored on a five‐point scale. The training was designed to make medical physicists effective communicators with patients, including instruction on how to tailor the information to the specific patient.

Patients were chosen to participate in the physicist–patient consult program as part of a pilot study (randomly selected from participating physician services), a clinical trial (available to all patients), or an ad hoc request (by the attending physician, radiation therapist, or patient). The only requirements for participation were that the patient be receiving, or scheduled for, external beam radiation therapy and fluent in the English language.

Each patient received two physicist–patient consults. The first physicist–patient consult took place immediately prior to the computed tomography (CT) simulation appointment, and the second physicist–patient consult took place immediately prior to the first treatment appointment. A single physicist–patient consult occurred when patients or staff requested an ad hoc meeting with a physicist outside of the standard physicist–patient consult program.

In the first physicist–patient consult, the medical physicist explained the role of a medical physicist and provided an overview of the CT simulation, treatment planning, and treatment delivery processes. During this meeting, the medical physicist also explained that they would be the primary resource for all of the technical aspects related to the patient’s radiation therapy treatment.

**Fig. 1:**
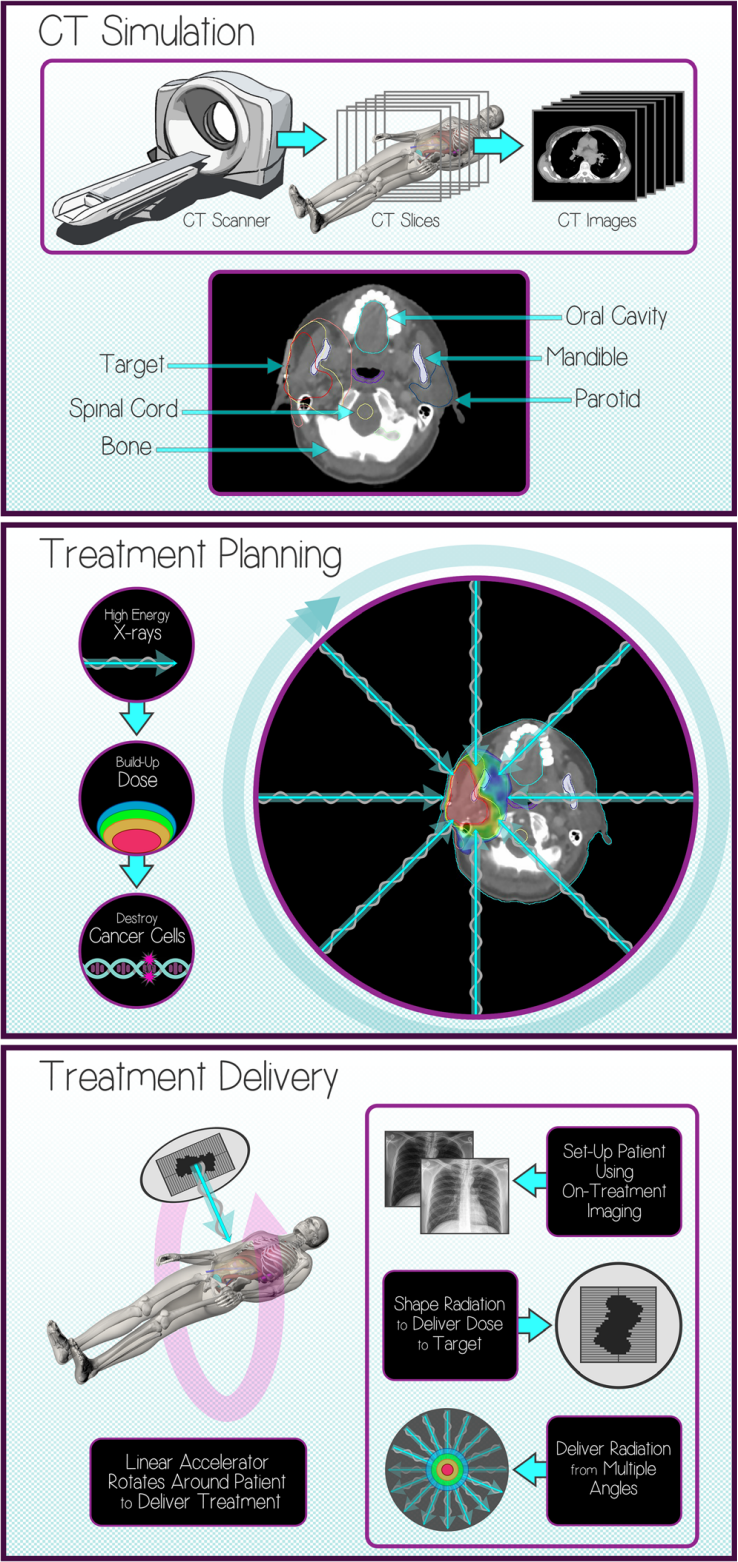
Examples of the three infographics created for the physicist–patient consults. The first infographic (top) describes the computed tomography (CT) simulation process (personalized by inserting a CT image of the patient and labeling the target and normal tissues). The second infographic (middle) describes the treatment planning process (personalized by inserting a CT image of the patient and illustrating the dose distribution and beam angles). The third infographic (bottom) describes the treatment delivery process (the same for all patients).

In the second physicist–patient consult, the same medical physicist from the first consult met with the patient to provide information about the patient’s specific treatment plan and treatment delivery technique. For this consult, three infographics were created for the patient. These educational materials were used to guide the discussion, ensure that all of the relevant topics were covered, and help facilitate patient questions. The first infographic was used to explain how the CT simulation images were used to delineate the target and normal tissues. This infographic was personalized for the patient by including an image from the patient’s CT scan, with the target and relevant normal tissues labeled. The second infographic was used to describe how various beam angles or arcs were utilized to deliver the prescription dose to the target, while avoiding unnecessary dose to the surrounding normal tissues. This infographic was personalized for the patient by including an image from the patient’s CT scan, with the dose colorwash and beam angles overlaid. The third infographic was designed to explain the treatment delivery process, including when and how imaging will be utilized to align the patient before treatment and how the linear accelerator shapes the treatment beam to deliver the radiation. This infographic was the same for all patients. Ancillary devices used for respiratory motion management or surface image guidance were also discussed during this physicist–patient consult, when applicable. Creating the personalized infographics for each patient helped standardize the physicist–patient consult process. The infographics ensured that every patient received the same information about their radiation treatment, hopefully mitigating any potential cognitive biases on the part of the physicists.^14^ Examples of the three infographics are illustrated in Fig. 1. A complete overview of the physicist–patient consult program (including the design, implementation, and specific objectives) and the metrics used to assess patient anxiety and satisfaction are described in detail elsewhere.^11^

In the initial months of the study (August 2016–December 2017), following each physicist–patient consult, all patient questions were documented by the physicists. The questions were compiled into a comprehensive list. For the remaining time period (January 2018–December 2019), any newly encountered questions were periodically added to the list. Patients often asked the same question in different ways, so similar questions were combined into a single question where appropriate. One hundred fifty‐two physicist–patient consults were included from August 2016 to December 2019. The physicists participating in the physicist–patient consult program reviewed the questions together for two purposes: (a) to combine similar questions, and (b) to determine a number of topical categories to further group questions. An example of two questions that were combined into the same question is “Are there different types of radiation?” and “Is all radiation the same?”. Therefore, the number of questions grouped into topical categories will be less than the total number of questions asked during the 152 patient consults.

## 3 RESULTS

The patient cohort included multiple external beam radiation therapy treatment sites, palliative and curative intents, stereotactic body radiation therapy (SBRT) and stereotactic radiosurgery (SRS) techniques, and respiratory motion management and surface image guidance ancillary devices. A total of 88 unique questions were asked during the 152 physicist–patient consults. The first consult (prior to CT simulation) required approximately 15 min of preparation time and approximately 15 min of time with the patient, and the second consult (prior to the first treatment) required approximately 1 h of preparation time and approximately 30 min of time with the patient. The majority of the preparation time for the first consult was devoted to reviewing the patient’s chart. The majority of preparation time for the second consult was devoted to reviewing the patient’s treatment plan and creating the personalized infographics. On average, patients asked more questions during the second physicist–patient consult (prior to the first treatment) than the first physicist–patient consult (prior to the CT simulation).

Grouping the questions resulted in four topical categories: (a) Treatment Planning and Delivery Questions, (b) General Radiation Questions or Concerns, (c) Safety and Quality Assurance Questions, and (d) Medical Questions. The number and percentage of patient questions in each category after similar questions were combined into a single question, along with five questions that were frequently asked during physicist–patient consults in each category, are shown in Table 1. The Dataset S1 contains the complete list of patient questions, also after similar questions were combined into a single question.

**Table 1:** Patient question categories and common questions

Category (number of questions, percent of total)	Common Questions
Treatment Planning and Delivery Questions (54, 61.4%)	What type of radiation am I getting? Is my treatment plan customized for me? How is my treatment plan created? Does the radiation go everywhere or just to my tumor? How does the treatment machine work?
General Radiation Questions or Concerns (15, 17.1%)	Are there different types of radiation? Can radiation cause cancer? Will radiation make me radioactive? How does radiation kill tumor cells? How does the body dispose of the tumor cells after they die?
Safety and Quality Assurance Questions (13, 14.8%)	How do you know the treatment machine is delivering the correct dose? Do you check my status as I go through treatment? How often does something go wrong during treatment? Does anyone check the treatment machine? Has anyone else reviewed my treatment plan to make sure it’s correct?
Medical Questions (6, 6.8%)	What kind of side effects can I expect? When will I start to feel the side effects? When will I start to notice a difference from the treatment? Can I continue eating/taking [*insert any number of foods/supplements*]? Can I continue [*insert any number of activities*]?

## 4 DISCUSSION

More questions fell into the “Treatment Planning and Delivery Questions” category than the other three categories combined. This may be due to the standardized process of the physicist–patient consults and the personalized infographics. Both of these guided the conversations and appeared to help patients form questions targeted at this topic. Patients were most interested in understanding how their treatment was customized for their disease and what was being done to limit radiation to normal healthy tissues.

The infographics provided a way to facilitate questions from the patients. Some patients came into the consults with a list of questions they wanted to ask. Others were slow to ask questions, until they became comfortable in the consult. Often patients would initially comment that they did not have any questions, but once they saw the personalized infographics, they began asking questions.

The fewest number of patient questions were in the “Medical Questions” category. This was expected because during the initial physicist–patient consult with each patient, the differences between a medical physicist and a radiation oncologist were clearly articulated. This introduction helped to ensure that patient questions were related to the technical aspects of treatment. However, when medical questions did arise, they were most frequently related to potential side effects. Patients appeared to be comfortable with the physicist not answering the medical aspects of those questions, while informing the patient that the physicist will bring the question to the attending radiation oncologist for follow‐up at their next meeting (e.g., weekly on‐treatment visit) or by a phone if the question was urgent.

One limitation is that the results of this study were not controlled for patient demographics or socioeconomic factors. Although information such as community type (rural vs urban) or health literacy could potentially be beneficial in predicting what types of questions patients ask, it was beyond the scope of this study. This could be a topic for future work.

## 5 CONCLUSION

Physicist–patient consults provided an opportunity to address the technical aspects of radiation therapy with patients in greater detail. The fact that patient questions could be conveniently grouped into only four topical categories indicates that it may be straightforward for other medical physicists to prepare for effectively addressing technical questions during physicist–patient consults.

## CONFLICT OF INTEREST

No conflict of interest.

## Supporting information


**Dataset S1**. Complete list of patient questions.
