# Supplementary material for: A review of patient questions from physicist—patient consults
Source: J Appl Clin Med Phys. 2020 Jun 9;21(8):305–8. doi: 10.1002/acm2.12942 (PMC7484844; doi:10.1002/acm2.12942)
Supplement: Supplementary file 1 — Dataset S1. Complete list of patient questions. [file ACM2-21-305-s001.docx]

**Supplemental Material: Complete List of Patient Questions**

**General Radiation Questions or Concerns**

1. What is radiation?
2. Are there different types of radiation?
3. What is background radiation?
4. Does [*insert household device*] produce radiation?
5. Can radiation cause cancer?
6. Will radiation make me radioactive?
7. Can you see radiation?
8. Can you feel radiation?
9. How does radiation work?
10. How does radiation kill tumor cells?
11. How does the body dispose of the tumor cells after they die?
12. What does ‘ionizing’ mean?
13. Does all of the radiation I receive build-up over time?
14. I’ve had radiation before, do I need to be worried?
15. If my cancer comes back, can we treat it again?

**Treatment Planning and Treatment Delivery Questions**

1. Am I being treated today (at CT simulation)?
2. What type of radiation am I getting?
3. What is my prescription; how does it compare to others?
4. Is my treatment plan customized for me?
5. How is my treatment plan created?
6. Who makes my treatment plan?
7. How do you know what to treat?
8. How do you know where the tumor is in my body?
9. How is the radiation shaped?
10. How is the dose shaped to my tumor?
11. How do you know what dose to give the tumor?
12. Does the radiation go everywhere or just to my tumor?
13. Why is the target larger than the tumor?
14. How does the treatment dose compare to the radiation I get in normal life?
15. How do you know what doses my healthy organs can tolerate?
16. How do you spare the [*insert organ*]?
17. The treatment machine is pointed right at me, won’t the radiation go into my heart
    and lungs?
18. How do you know the treatment is working?
19. What happens if I move during treatment?
20. What happens when I breathe during treatment and the tumor moves?
21. How do you know I don’t need to take a deep breath during treatment (for a free-breathing patient)?
22. How do you know the cancer hasn’t changed between CT simulation and treatment?
23. How do you know if the tumor has changed size?
24. Can you see if the tumor is getting smaller during treatment?
25. If the tumor shrinks during treatment, will the plan be delivering the wrong dose?
26. How much radiation am I getting from set-up imaging?
27. How does the x-ray imaging dose differ from the treatment dose?
28. Why did I have more set-up images taken today than yesterday?
29. I’m afraid of the dose from the imaging every day, can I forgo having this?
30. Why do I need a full bladder?
31. How long will the treatment take?
32. Why does the treatment machine move around me during treatment?
33. Why does the collimator move during treatment?
34. Is this a new type of treatment machine?
35. Does the radiation stop after you turn the machine off?
36. Are all the treatment machines the same?
37. What are the sounds I’m hearing during treatment?
38. Will I feel anything during treatment?
39. Will I feel an electric shock during treatment?
40. How does the treatment machine work?
41. What *about* [*insert another type of therapy or machine*], is that better?
42. Why does treatment take so many days?
43. Why is the dose spread out over so many days?
44. Why has my number of treatments changed?
45. I would like to delay my treatment; can we do this at a later date?
46. Why should I not skip a day of treatment?
47. If it’s important that I not skip a treatment, then why don’t you treat on the weekend?
48. Why am I getting a boost with a different type of treatment?
49. Will the gold fiducials become radioactive as I’m treated?
50. Why was my friend’s radiation for the same disease site different from mine?
51. What is dose?
52. What is a gray?
53. What does IMRT mean?
54. What does SBRT mean?

**Safety and Quality Assurance Questions**

1. How do you know the treatment machine is delivering the correct dose?
2. How accurately can you deliver the radiation?
3. How accurately is the dose delivered?
4. Has anyone else reviewed my treatment plan to make sure it’s correct?
5. Do you check my status as I go through the treatment?
6. How do you know if something went wrong during the treatment?
7. How often does something go wrong during the treatment?
8. Is it okay that I was transferred to a different treatment machine for treatment?
9. Why is/was the treatment machine down?
10. How do you know it’s safe for me to start treatment again (after the machine was down)?
11. This is a new machine; how do you know it works properly?
12. Does anyone check the treatment machine?
13. How often do you perform the [*insert type of quality assurance*]?

**Medical Questions**

1. What kind of side effects can I expect?
2. When will I start to feel the side effects?
3. When will I start to notice a difference from the treatment?
4. Can I continue [*insert any number of activities*]?
5. Can I continue eating/taking [*insert any number of foods/supplements*]?
6. Can I use [*insert any medication/ointment*] on my breast (breast cancer patient)?
